# Unveiling the Genomic Landscape of G2P[6] Rotavirus a Strains in Brazil: Evolutionary and Epidemiological Perspectives

**DOI:** 10.3390/v17081103

**Published:** 2025-08-11

**Authors:** Vanessa Cristina Martins Silva, Yasmin França, Lais Sampaio de Azevedo, Raquel Guiducci, Edlaine Faria de Moura Villela, Adriana Luchs

**Affiliations:** 1 Virology Center, Adolfo Lutz Institute, Sao Paulo 01246-902, Brazil; 2 Graduate Program in Sciences of the Center for Disease Control, São Paulo State Department of Health, Sao Paulo 01246-000, Brazil

**Keywords:** molecular surveillance, gastroenteritis, Genetic diversity, rotavirus, public health

## Abstract

In Brazil, molecular surveillance expanded after Rotarix™ vaccine introduction, alongside G2P[4] dominance. The G2P[6] genotype, despite sharing the same DS-1-like constellation as G2P[4] strains, remains rare. This retrospective study analyzed eight Brazilian G2P[6] strains (2012–2014) through RT-PCR and 11-segments sequencing, followed by phylogenetic analysis. Two distinct groups were identified: 2012–2013 strains (six) carried a DS-1-like backbone with the rare NSP4 E6 genotype, while 2014 strains (two) exhibited the classical DS-1-like constellation with E2. Phylogenetic analysis confirmed the two main clusters: 2012–2013 strains related to classical G2P[4] and uncommon global genotypes, and 2014 strains resembling emerging DS-1-like G1/G3/G8P[8] reassortants. The 2012–2013 strains clustered within G2-VP7 Lineage IVa, while the 2014 strains belonged to Lineage V, reflecting the global distribution of these variants. All VP4 genes were classified within the P[6]-Ia lineage, with phylogenetic analyses suggesting separate introductions from Asia and Africa. The E6 NSP4 gene segment identified in these strains has an undetermined origin and was not previously associated with G2P[6] strains in Brazil. Despite similarities to G2P[4], G2P[6] strains remain rare, with no genomic features explaining their limited spread. Phylogenetic data indicate multiple reassortment events and international viral exchange, highlighting Brazil’s role in RVA diversity. Ongoing full-genome surveillance is crucial to track rare variants and assess their public health relevance.

## 1. Introduction

*Rotavirus alphagastroenteritidis* (https://ictv.global/ accessed on 4 July 2025), also known as Rotavirus A (RVA), is a major cause of acute diarrhea in young children worldwide and also affects a broad spectrum of animal species [1,2]. It belongs to the genus *Rotavirus*, within the subfamily *Sedoreoviridae*, the family *Reovirales*, and the realm *Riboviria* [3]. Its genome consists of eleven segments of double-stranded RNA (dsRNA), encoding six structural proteins (VP1–VP4, VP6 and VP7) and five or six non-structural proteins (NSP1–NSP5/6) [4]. Traditionally, RVA strains were classified using a binary system based on the outer capsid proteins VP7 and VP4, referred to as G and P genotypes, respectively [5]. The genotypes G1P[8], G2P[4], G3P[8], G4P[8], G9P[8] and G12P[8] represent the six most prevalent strains found in humans [5,6].

To enable a more comprehensive genomic characterization, the RVA classification system was expanded to include all 11 genome segments, with the current nomenclature assigning specific genotypes to each segment: Gx–P[x]–Ix–Rx–Cx–Mx–Ax–Nx–Tx–Ex–Hx [7,8]. Most human RVA strains fall into one of three major genome constellations: the Wa-like genogroup 1 (Gx–P[x]–I1–R1–C1–M1–A1–N1–T1–E1–H1), the DS-1-like genogroup 2 (Gx–P[x]–I2–R2–C2–M2–A2–N2–T2–E2–H2) and, less commonly, the AU-1-like genogroup 3 (Gx–P[x]–I3–R3–C3–M3–A3–N3–T3–E3–H3) [7,8]. Following the introduction of the RVA genome constellation classification, whole-genome analyses of all 11 gene segments have revealed the virus’s genetic diversity, shaped by point mutations, gene rearrangements, inter- and intra-species reassortment and, to a lesser extent, homologous recombination [7,9].

Brazil has been conducting a genotypic surveillance of RVA strains for several decades, with efforts significantly intensified after the introduction of the Rotarix™ vaccine into the national immunization program in 2006 [6,10,11,12,13,14]. Afterward this implementation, a marked increase in the detection of the G2P[4] genotype was observed [6,15,16,17]. This phenomenon, of global significance, was also reported in multiple countries subsequent to the launch of universal RVA vaccination and initially led to the hypothesis that it might be associated with vaccine-driven selective pressure [18,19,20,21,22]. However, studies conducted thereafter, including those in countries without RVA vaccine coverage, suggest that more complex mechanisms related to viral ecology may be involved [23,24,25,26,27]. Between 2007 and 2010, the G2P[4] predominated throughout Latin America and the Caribbean, underscoring its high dissemination capacity [28].

In contrast, the G2P[6] genotype did not emerge widely in either vaccinated or non-vaccinated regions, remaining rare in human populations, despite sharing a DS-1-like backbone genotype constellation resembling that of G2P[4] [6,14,29,30,31,32,33,34,35,36]. This genotype exhibited an uneven pattern of emergence, being detected at low frequencies in various parts of the world, including Sub-Saharan Africa, Asia and Latin America [6,14,29,31,34,35,37]. Studies indicate that different lineages of the P[6] genotype may have originated from multiple interspecies reassortment events involving RVA from different animal hosts, accounting for the significant genetic heterogeneity observed in this genotype [38,39,40]. This association with zoonotic events and its predominance in neonatal infections reinforce the complexity of its transmission dynamics, a trend that has also been documented in Brazil [41,42,43,44,45].

The number of available full genome sequences for G2P[6] remains limited, largely due to its low prevalence [33,35]. To better understand the factors that limit the selective advantage of G2P[6] compared to G2P[4], it is essential to perform a complete genome sequencing of these strains in order to identify potential selective pressures influencing their spread. The aim of this study was to perform a molecular characterization and phylogenetic analysis of the 11 genome segments of eight Brazilian G2P[6] RVA strains identified between 2007 and 2014, to enhance understanding of the complex transmission dynamics and evolutionary fitness of this rare genotype. The findings are expected to contribute to a better understanding of viral ecology and support improved surveillance efforts in Brazil and beyond.

## 2. Material and Methods

### 2.1. Study Design and Sample Selection

This retrospective study analyzed convenience fecal samples from individuals of all age groups, collected through Brazil’s national viral gastroenteritis surveillance program, between 2007 and 2014. Sample processing was carried out at the Enteric Diseases Center (NDE) of the Adolfo Lutz Institute (IAL), a macro-regional reference center responsible for monitoring viral gastroenteritis in the Midwest and parts of the Southeast and South regions, under the coordination of the Ministry of Health (MS). For this study, all samples that tested positive for the G2P[6] genotype during routine surveillance at IAL were selected for further analysis. Specifically, eight G2P[6] strains were included: five strains identified in 2012 [6], one in 2013 and two in 2014 [34], originating from the states of São Paulo (Southeast region) and Paraná (South region) (Table 1).

### 2.2. Electropherotyping Analysis

RVA migration patterns were assessed by polyacrylamide gel electrophoresis (PAGE), followed by silver staining for visualization, as described by Herring et al. (1982) [46].

### 2.3. Whole-Genome Constellation Profiling

RVA dsRNA was extracted from 10% fecal suspensions using the Quick-DNA/RNA™ Viral MagBead Kit (Zymo Research^®^, Orange, CA, USA), according to the manufacturer’s instructions. RT-PCRs targeting the 11 genome segments were performed in-house using the following published primers: NSP1 [47,48,49]; NSP2, NSP4 and VP6 [48,49]; NSP3 and NSP5/6 [49]; VP1, VP2 and VP3 [50]; VP4 [51]; and VP7 [49,52]. Amplification conditions followed the protocol described by Gouvea et al. (1990) [52].

PCR products were resolved on 1.5% agarose gels stained with GelRed™ (Biotium, Fremont, CA, USA), alongside a 100 bp molecular-size ladder, and visualized using a gel documentation system. Amplicons were subsequently sequenced using the BigDye™ Terminator v3.1 Cycle Sequencing Kit (Applied Biosystems, Foster City, CA, USA), with the same primers used for PCR. Sequencing was carried out on an ABI 3500 Genetic Analyzer (Applied Biosystems) at the Premium Network of Multi-User Equipment/IMT/FMUSP. Chromatograms were manually edited using Sequencher™ 4.7 software (Gene Codes Corporation, Ann Arbor, MI, USA). Genotype assignment was performed using the Rotavirus A Genotyping Tool v0.1 (https://mpf.rivm.nl/mpf/typingtool/rotavirusa/ accessed on 1 November 2024).

### 2.4. Phylogenetic Inference

Sequences obtained in this study were aligned with reference RVA sequences for the genes NSP1–NSP5/6 and VP1–VP4, VP6 and VP7 retrieved from GenBank. Alignments were performed using the CLUSTAL W algorithm implemented in BioEdit v7.0.5.2 (Ibis Therapeutics, Carlsbad, CA, USA). Maximum likelihood (ML) phylogenetic trees were constructed for each gene segment using MEGA X [53], and the best-fitting nucleotide substitution models were selected according to the corrected Akaike Information Criterion (AICc). The following models were applied: T92+G+I for NSP1 (A2), VP1 (R2), VP3 (M2) and VP7 (G2); GTR+G+I for NSP2 (N2), T92+G for NSP3 (T2), NSP4 (E2) and NSP5 (H2); T92+I for NSP4 (E6); TN93+G for VP2 (C2); and HKY+G+I for VP4 (P[6]) and VP6 (I2). Branch support was assessed with 1000 bootstrap replicates.

Reference strains were included for lineage assignment based on previously published studies: Agbemabiese et al. (2019) [54] and Azevedo et al. (2023) [55] for DS-1-like constellations, Gupta et al. (2021) [56] for VP4 P[6] and Doan et al. (2015) [57] for VP7. Pairwise nucleotide identity was calculated using distance matrices in MEGA X [53].

## 3. Results

### 3.1. Genome Constellations

The G2P[6] RVA strains exhibited the characteristic short electropherotype associated with P[6] genotypes. Near-complete nucleotide sequences were obtained for the NSP1–NSP5/6, VP7 and VP6 segments, and partial sequences for the VP1–VP4 genes of eight G2P[6] strains. An exception was strain IAL-R1175/2012, for which only a partial NSP1 sequence could be obtained. The percentage of the genome sequenced among the strains ranged from 47.4% to 59.2%. The sequence lengths and the nucleotide positions analyzed for the eight G2P[6] strains are detailed in Table S1.

The two G2P[6] strains detected in 2014 (IAL-R50 and IAL-R52) exhibited the classical DS-1-like genomic constellation (I2-R2-C2-M2-A2-N2-T2-E2-H2), while the five strains from 2012 (IAL-R1000, IAL-R1007, IAL-R1142, IAL-R1175 and IAL-R1228) and the single strain from 2013 (IAL-R126) presented a DS-1-like backbone associated with the rare E6 genotype in the NSP4 segment (I2-R2-C2-M2-A2-N2-T2-E6-H2) (Table 1).

### 3.2. Analysis of the Outer Capsid Glycoprotein VP7

**Figure 1:**
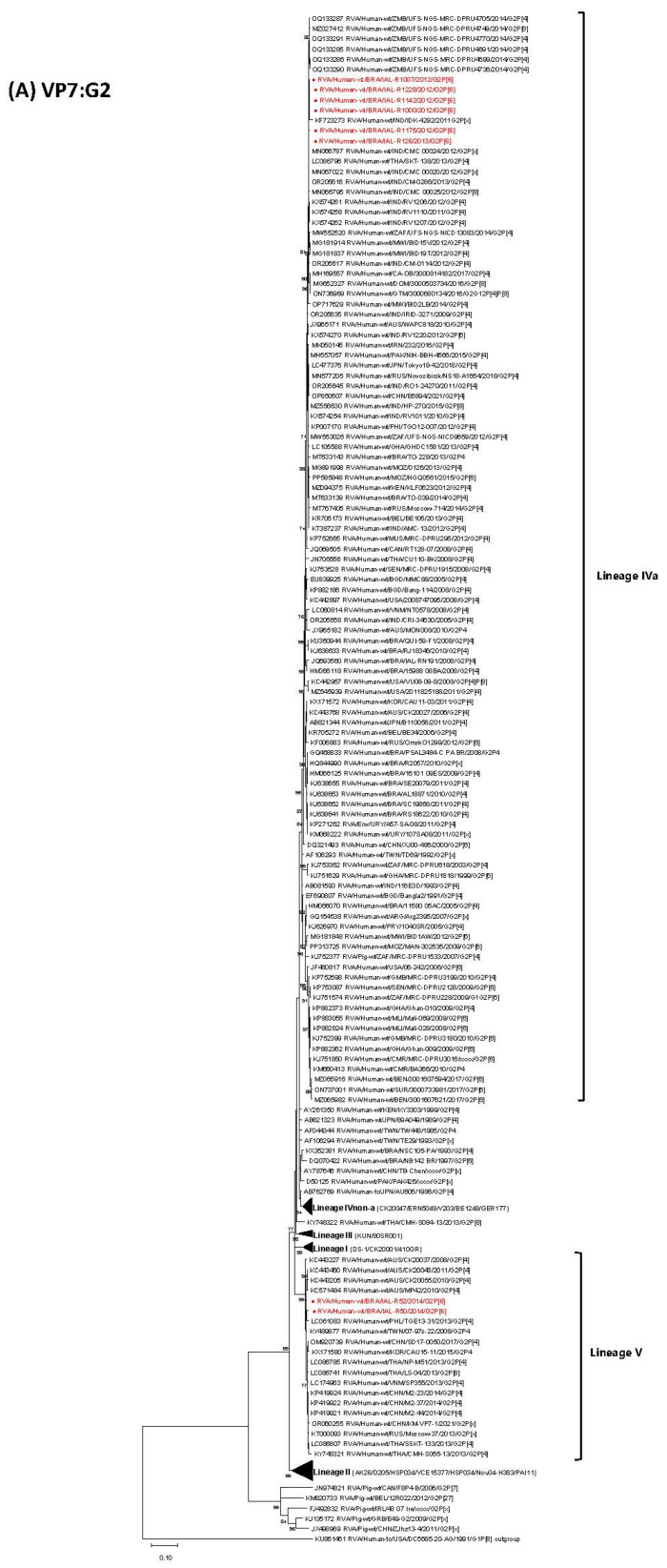

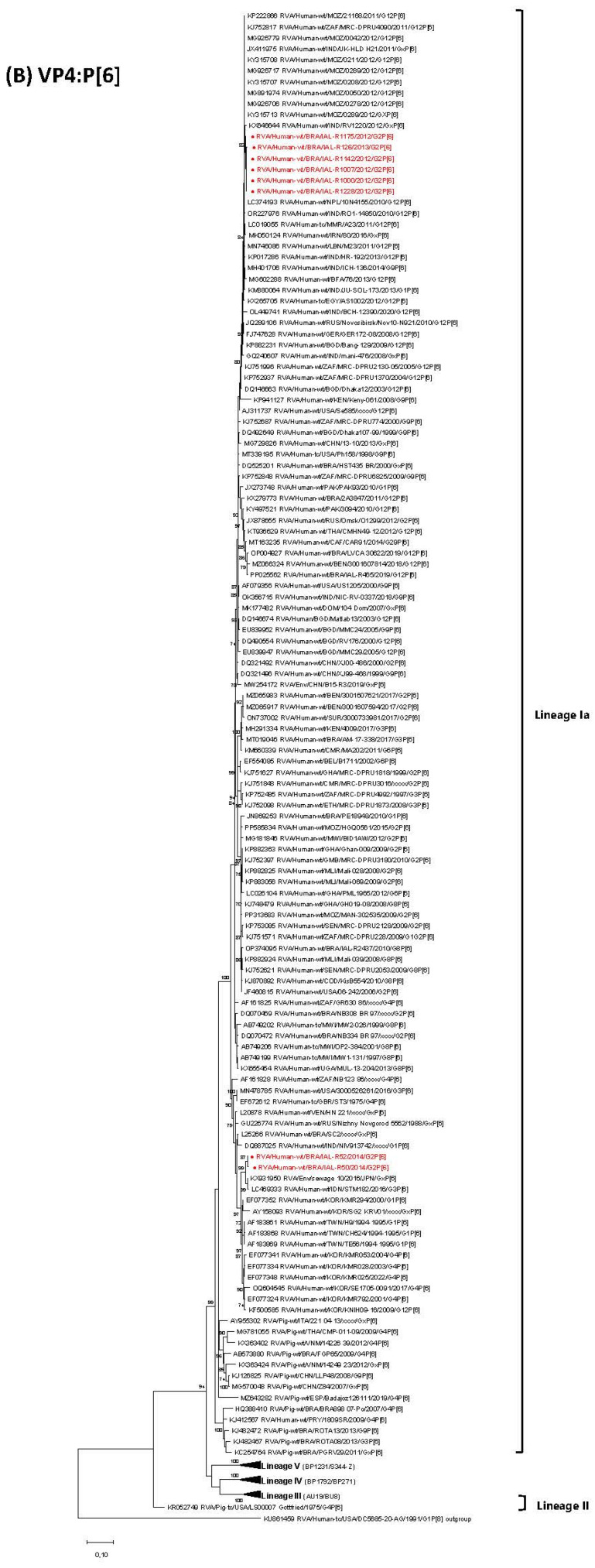

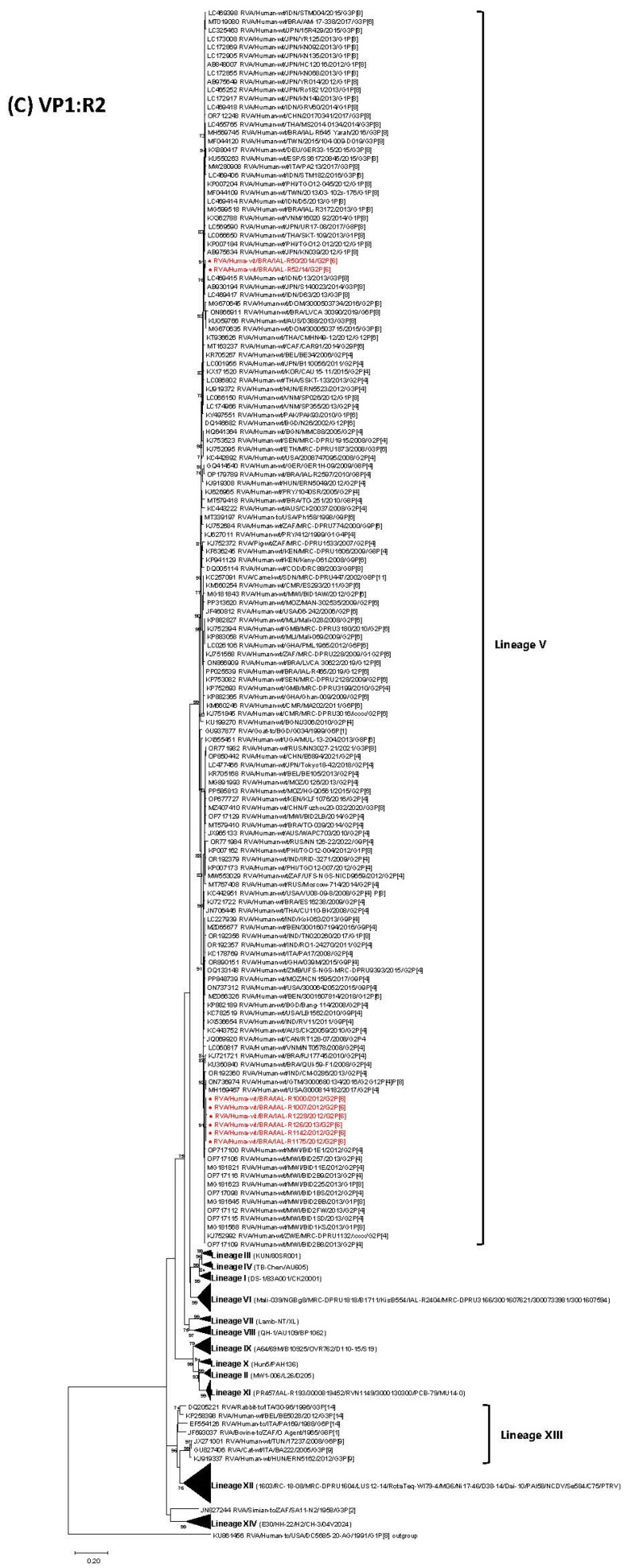

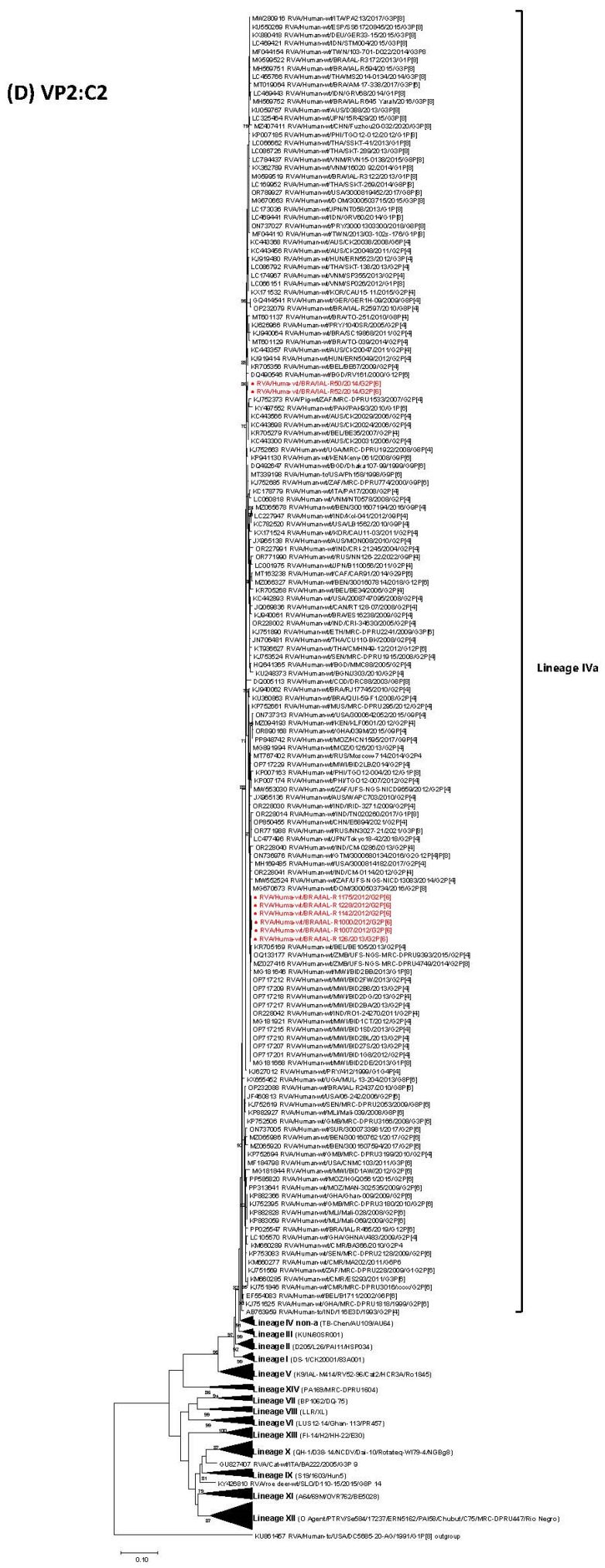

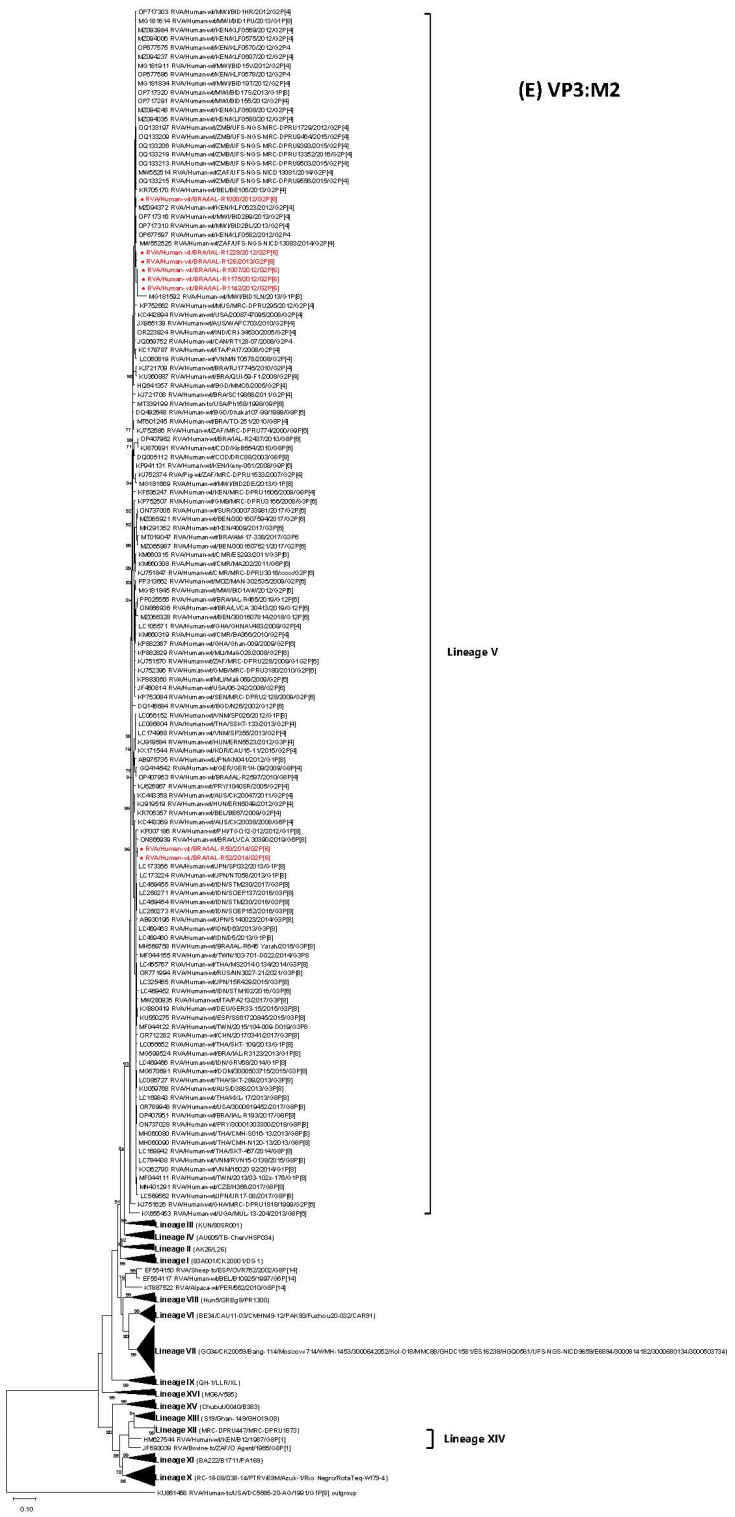

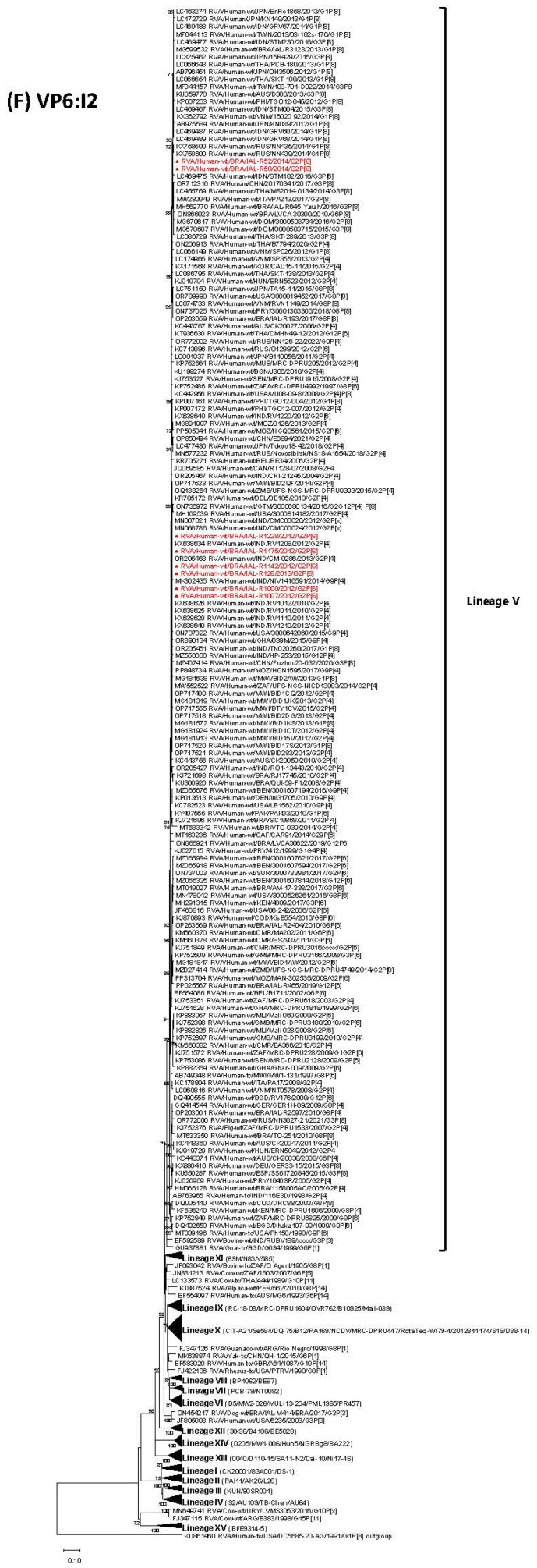

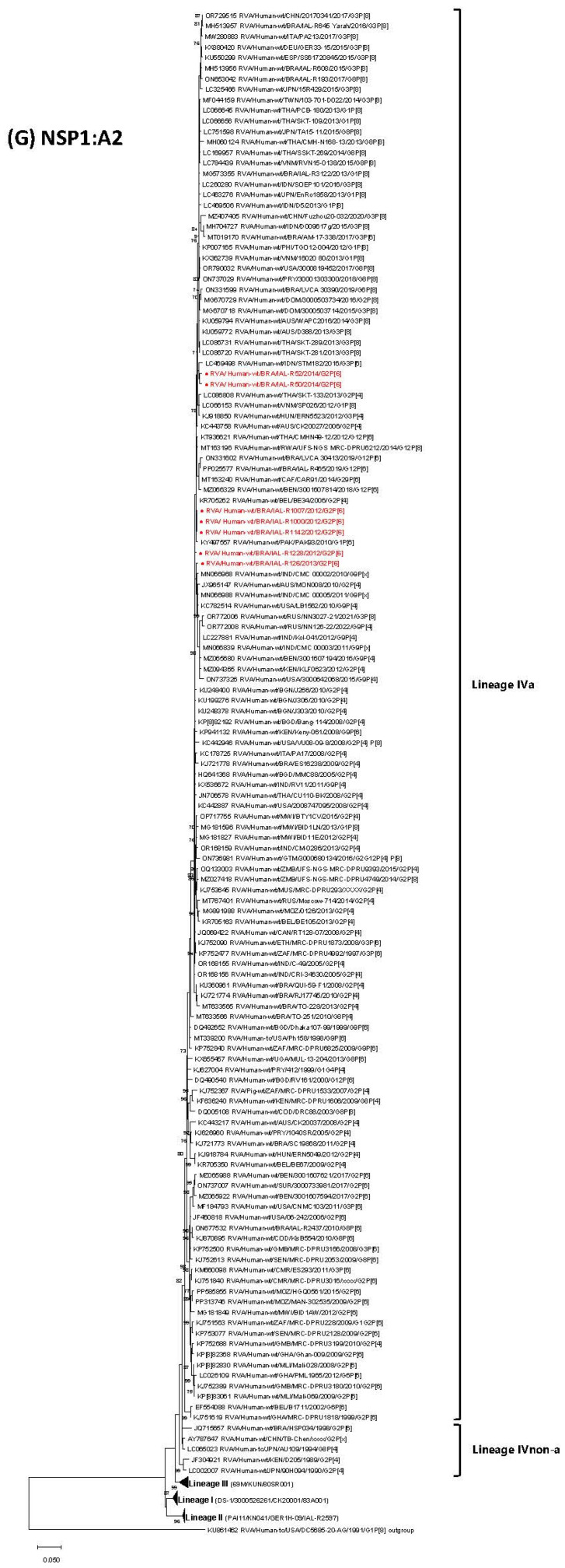

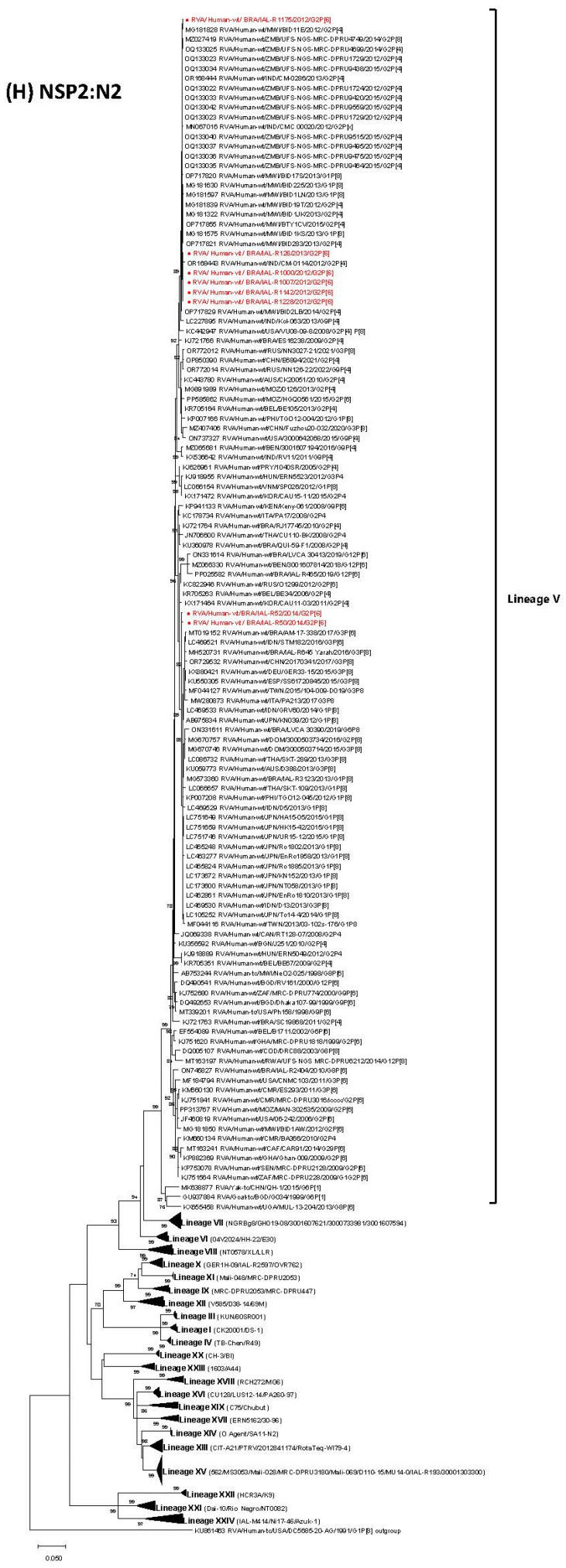

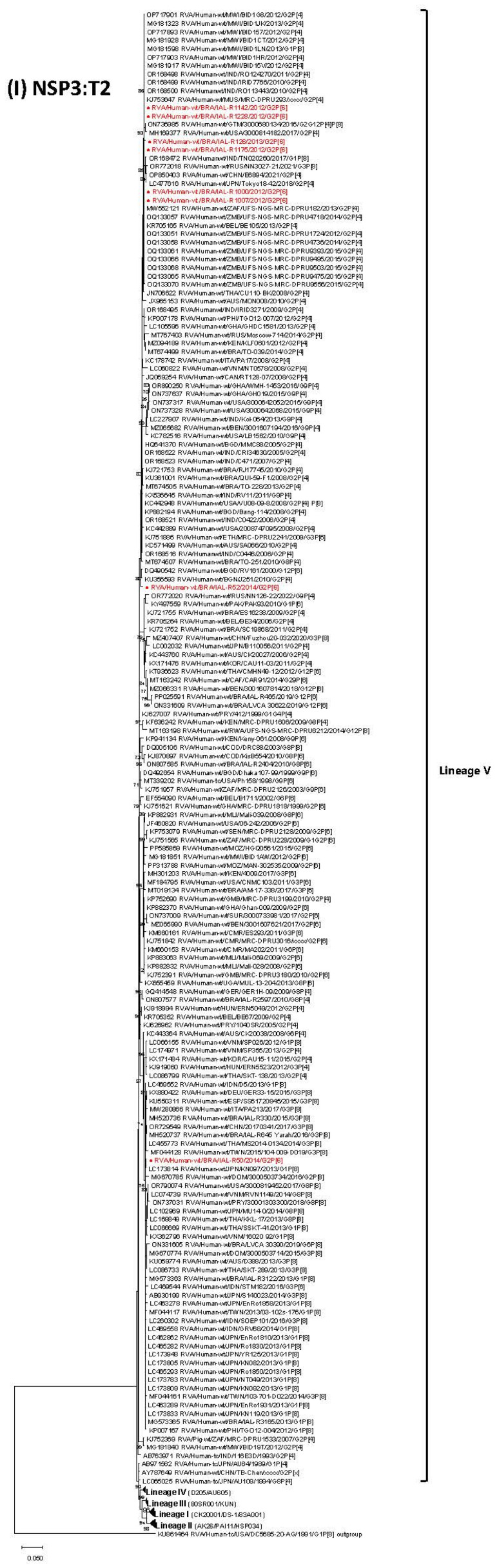

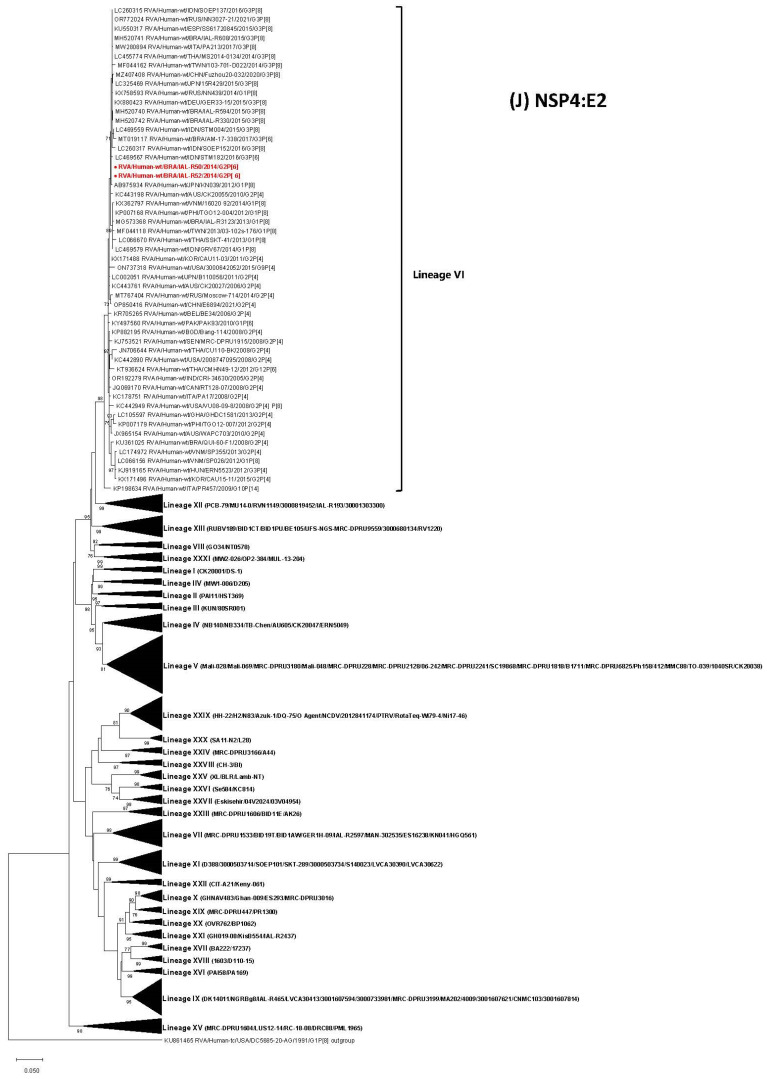

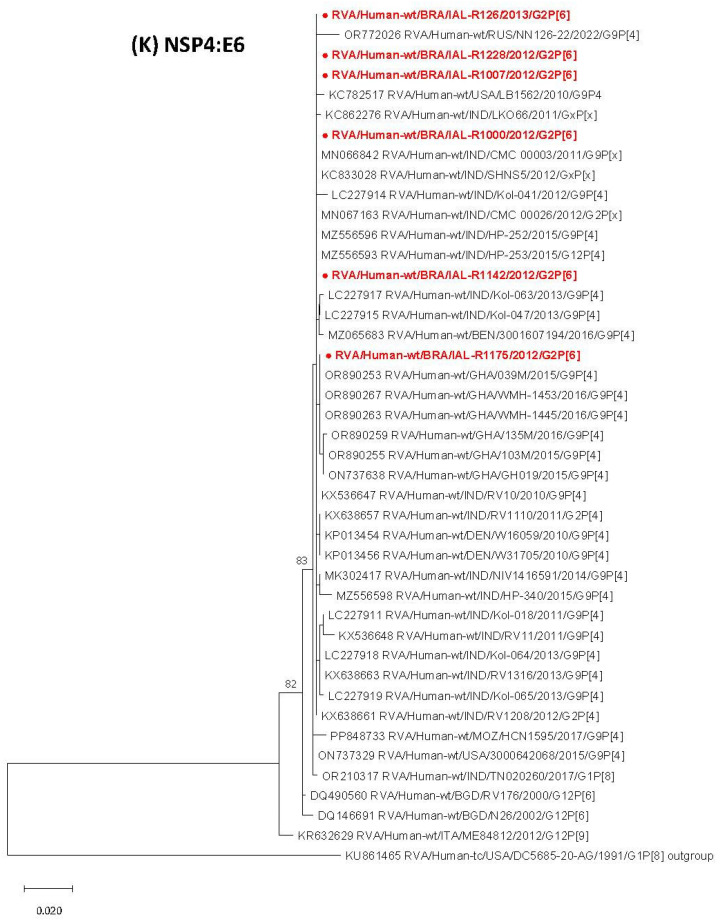

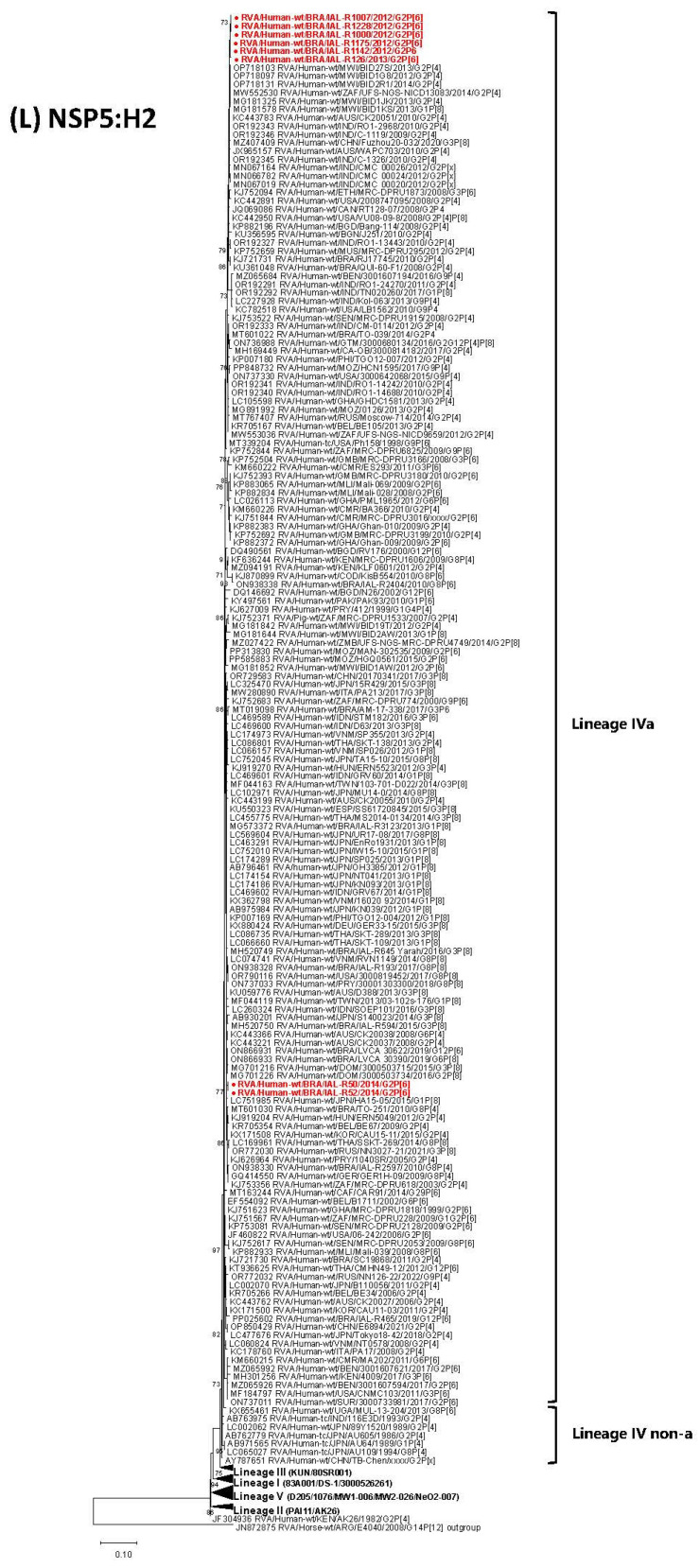
Maximum likelihood phylogenetic trees based on nucleotide sequences of 11 genome segments [VP7-G2 (**A**), VP4-P[6] (**B**), VP1-R2 (**C**), VP2-C2 (**D**), VP3-M2 (**E**), VP6-I2 (**F**), NSP1-A2 (**G**), NSP2-N2 (**H**), NSP3-T2 (**I**), NSP4-E2 (**J**), NSP4-E6 (**K**) and NSP5/6-H2 (**L**)] were constructed using MEGA X software to assess the genetic relatedness of eight Brazilian G2P[6] RVA strains (IAL-R1007/2012, IAL-R1000/2012, IAL-R1228/2012, IAL-R1142/2012, IAL-R1175/2012, IAL-R126/2013, IAL-R52/2014 and IAL-R50/2014; shown in bold red) to global reference strains, with bootstrap values indicated at nodes and scale bars representing nucleotide substitutions per site.

Globally representative strains from the five known G2 lineages (I, II, III, IVa, IVnon-a and V) were included in this analysis. The Brazilian G2P[6] strains analyzed in this study clustered separately. Strains IAL-R1007/2012, IAL-R1000/2012, IAL-R1228/2012, IAL-R1142/2012, IAL-R1175/2012 and IAL-R126/2013 grouped within Lineage IVa, exhibiting nucleotide identities ranging from 99.9% to 100%. In contrast, strains IAL-R52/2014 and IAL-R50/2014 were classified within Lineage V, sharing 99.5% nucleotide identity with each other (Table 1, Figure 1A).

Strains from Lineage IVa (IAL-R1007/2012, IAL-R1000/2012, IAL-R1228/2012, IAL-R1142/2012, IAL-R1175/2012 and IAL-R126/2013) exhibited a high nucleotide identity (99.0–100%) with classical G2P[4]/G2P[x] strains detected between 2009 and 2017 in geographically diverse areas, including India, Thailand, Australia, Zambia, Malawi, South Africa, the United States, Pakistan, Ireland and Mauritius. They also demonstrated close similarity (99.2–99.8% nt) to atypical genotypes, such as G2G12P[4]P[8] and G2P[8], reported in India, Zambia, the Dominican Republic and Guatemala between 2012 and 2016. Conversely, these Lineage IVa strains were more distantly related to earlier Brazilian G2P[4]/G2P[x] strains identified across the country, such as 11580_05AC/2005 (95.6–95.9% nt), IAL-RN191/2008 (96.6–96.9% nt), 15988_08BA/2008 (97.5–97.9% nt), QUI-59-F1/2008 (97.2–97.5% nt), PSAL3484-C_PA_BR/2008 (97.1–97.3% nt), 16101_09ES/2009 (97.1–97.4% nt), AL18871/2010 (97.4–97.6% nt), R2057/2010 (97.2% nt), RJ18346/2010 (96.9–97.3% nt), RS18622/2010 (97.1–97.4% nt), SE20079/2011 (97.3–97.5% nt), SC19868/2011 (97.1–97.4% nt), TO-228/2013 (98.2–98.8% nt) and TO-039/2014 (98.4–98.6% nt) (Figure 1A).

Strains IAL-R52/2014 and IAL-R50/2014 (Lineage V) were closely related to G2P[4]/G2P[x] strains detected between 2008 and 2021 in Taiwan, Australia, Thailand, China, South Korea, Vietnam, Russia and the Philippines, showing nucleotide identities of 98.2–99.3%. They also shared 99.1% nt identity with the atypical strain RVA/Human-wt/THA/LS-04/2013/G2P[8]. The RVA/Human-wt/BRA/NB142/1997/G2P[6] strain, identified in hospitalized neonates with community-acquired diarrhea during a G2P[6] outbreak in Belém, Pará, in 1997 [28,42], did not share a lineage with any of the Brazilian strains described herein (88–92.7% nt) (Figure 1A).

### 3.3. Analysis of the Spike Protein VP4

Regarding the VP4 gene, all eight Brazilian G2P[6] strains analyzed in this study clustered within Lineage P[6]-Ia, occupying two different branches. Strains IAL-R1000/2012, IAL-R1007/2012, IAL-R1142/2012, IAL-R1228/2012, IAL-R1175/2012 and IAL-R126/2013 exhibited high nucleotide identity (99.0–99.6%) with atypical G12P[6]/GxP[6]/G9P[6] strains detected in Africa (Mozambique; South Africa) and Asia (Myanmar; Nepal; India). On the other hand, strains IAL-R50/2014 and IAL-R52/2014 were genetically closer to Asian strains exclusively, encompassing RVA/Env/sewage_10/2016/JPN/GxP[6] (98.5–98.8% nt) and RVA/Human-wt/IDN/STM182/2016/G3P[6] (98.1–98.4% nt) (Table 1, Figure 1B).

Compared to previously reported Brazilian P[6] RVA strains, IAL-R1000/2012, IAL-R1007/2012, IAL-R1142/2012, IAL-R1228/2012, IAL-R1175/2012, IAL-R126/2013, IAL-R50/2014 and IAL-R52/2014 displayed greater genetic divergence, with lower nucleotide identities when aligned with HST435_BR/2000/GxP[6] (92.5–96.9% nt), 2A3847/2011/G12P[6] (90.7–95.6% nt), LVCA_30622/2019/G12P[6] (89.2–94.3% nt), IAL-R465/2019/G12P[6] (89.5–94.9% nt), AM-17–338/2017/G3P[6] (90.5–94% nt), PE18948/2010/G1P[6] (91.7–94.3% nt), IAL-R2437/2010/G8P[6] (91.2–94.8% nt) and SC2/xxxx/GxP[6] (92.7–95.8% nt). This divergence also extended to strains identified in neonatal patients from Belém, comprising RVA/Human-wt/BRA/NB334/1997/G2P[6] and RVA/Human-wt/BRA/NB308/1997/G2P[6] (92.3–97% nt) [42]. Moreover, the Brazilian IAL P[6] strains showed significant genetic differences from swine-origin P[6] strains (84.4–89.6% nt), including those previously reported in Brazil (84.5–89.5% nt) (Figure 1B).

### 3.4. Phylogenetic Analysis of VP1–VP3 and VP6

Phylogenetic analyses of the VP1, VP2, VP3 and VP6 gene segments revealed that the Brazilian strains IAL-R1000/2012, IAL-R1007/2012, IAL-R1142/2012, IAL-R1228/2012, IAL-R1175/2012 and IAL-R126/2013 clustered closely together, sharing high nucleotide identity (≥99.3 nt). These strains formed a distinct phylogenetic branch separate from the other two Brazilian G2P[6] RVA strains, IAL-R50/2014 and IAL-R52/2014, which also exhibited high mutual similarity (≥99.8% nt). Despite clustering separately, all strains were classified within the same lineages: R2-V, C2-IVa, M2-V and I2-V (Table 1, Figure 1C–F).

Comparative analysis revealed that the Brazilian strains IAL-R1000/2012, IAL-R1007/2012, IAL-R1142/2012, IAL-R1228/2012, IAL-R1175/2012 and IAL-R126/2013 exhibited significant nucleotide sequence similarity (≥99% nt) in the VP1, VP2 and VP3 gene segments when compared with classical G2P[4] strains possessing a DS-1-like genomic constellation reported worldwide between 2005 and 2021. These reference strains were broadly distributed across Africa, Europe, the Americas, Asia and Oceania (South Africa; Kenya; Mauritius; Mozambique; Malawi; Zambia; Zimbabwe; Belgium; Russia; USA; Canada; India; Bangladesh; China; Japan; Australia). Furthermore, the same Brazilian G2P[6] strains demonstrated notable genetic similarity (99–99.8% nt) in the VP1 and VP2 segments with atypical strains, including G9P[4], G12P[4]P[8], G2G12P[4]P[8] and G2P[8], identified from 2010 to 2017 in Mozambique, USA, India, Benin, Guatemala and the Dominican Republic. In addition, a high nucleotide identity (≥99% nt) in VP1, VP2 and VP3 genes was also observed between the Brazilian G2P[6] IAL-R1000/2012, IAL-R1007/2012, IAL-R1142/2012, IAL-R1228/2012, IAL-R1175/2012 and IAL-R126/2013 strains and emergent double-reassortant G1P[8] DS-1-like strains found in Malawi and the Philippines during 2012–2013 (Figure 1C–E).

The Brazilian G2P[6] strains IAL-R50/2014 and IAL-R52/2014 exhibited elevated nucleotide similarity in the VP1, VP2 and VP3 genes with emerging double-reassortant strains, including G1P[8] DS-1-like, equine-like G3P[8] DS-1-like and bovine-like G8P[8] DS-1-like genotypes detected worldwide, notably in Latin America and Asia. Specifically, the VP1 gene showed 99.0–99.5% nt identity with strains circulating between 2012 and 2017 in Asian countries (Japan; India; the Philippines; China; Taiwan; Vietnam; Thailand), as well as 99.1–99.2% nt identity with a Brazilian G1P[8] DS-1-like strain from 2013 (IAL-R3172; MG599518) (Figure 1C). Likewise, the VP2 gene exhibited 99.0–99.2% nt similarity with double-reassortant strains circulating from 2012 to 2018 in India, Thailand, Paraguay, USA, the Dominican Republic, Japan, Vietnam and Australia, in addition to 99.0% nt identity with the Brazilian G1P[8] DS-1-like (IAL-R3122; MG599519) and equine-like G3P[8] DS-1-like (IAL-R645_Yarah; MH569752) strains detected in 2013 and 2016, respectively. Furthermore, the VP2 gene also showed close relation (99.0–99.4% nt) to the G2P[4] and uncommon G8P[4]/G6P[4]/G9P[6]/G1G4P[4]/G3P[4] strains documented between 1999 and 2015 across Australia, Asia, Europe and Africa (Figure 1D). The VP3 gene also shared high identity (99.0–99.5% nt) with double-reassortant strains detected globally from 2013 through 2021 (Australia; India; the Czech Republic; Japan; Thailand; China; Taiwan; Vietnam; USA; the Dominican Republic; Germany; Spain; Russia; Paraguay), and 99.0–99.4% nt similarity with Brazilian G1P[8] DS-1-like (IAL-R3123; MG599524), equine-like G3P[8] DS-1-like (IAL-R645_Yarah; MH569758) and bovine-like G8P[8] DS-1-like (IAL-R193; OP407951) strains isolated from 2013 to 2017 (Figure 1E).

Finally, analysis of the VP6 gene revealed a binary clustering pattern among the Brazilian G2P[6] strains, dividing them into two distinct branches [strains 2012–2013 versus strains 2014] consistent with the distribution previously observed for other genomic segments (VP1–VP4 and VP7). The 2012–2013 strains exhibited high nucleotide identity (≥99.0% nt) with a broad range of classical G2P[4]/G2P[x] strains (2006–2018), atypical G2G12P[4]P[8]/G9P[4]/G12P[4]/G3P[6]/G12P[6] genotypes (1997–2017), including the RVA/Human-wt/RUS/O1299/2012/G2P[6] (99.2–99,4% nt), and emerging double-reassortant G1P[8] DS-1-like/equine-like G3P[8] DS-1-like strains (2013–2020) from Asia, Africa, Europe and the Americas. Similarly, the 2014 strains shared high similarity (99.1–99.8% nt) with double-reassortant G1P[8] DS-1-like and equine-like G3P[8] DS-1-like strains circulating between 2012 and 2017 across multiple regions, including Brazil (RVA/Human-wt/BRA/IAL-R3123/2013/G1P[8]; RVA/Human-wt/BRA/IAL-R645_Yarah/2016/G3P[8]) (99.4–99.8% nt). Despite this divergence, both clusters showed close nucleotide identity with a shared set of reference strains, including classical G2P[4] strains detected in Bangladesh and Senegal in 2008 and 2010, respectively (99.0–99.6% nt), as well as unusual strains, such as G3P[4] and G2P[4]P[8], recognized during the 2008–2012 period in Hungary and the USA (99.0–99.4% nt) (Figure 1F).

### 3.5. Phylogenetic Analysis of NSP1-NSP5 Genes

Consistent with the pattern observed for the structural proteins VP1–VP3 and VP6, phylogenetic analyses of the non-structural proteins NSP1, NSP2 and NSP5 likewise displayed a binary clustering trend. The Brazilian G2P[6] strains IAL-R1000/2012, IAL-R1007/2012, IAL-R1142/2012, IAL-R1228/2012, IAL-R1175/2012 and IAL-R126/2013 clustered together, exhibiting a nucleotide identity ranging from 99.0% to 100%, whereas strains IAL-R50/2014 and IAL-R52/2014 grouped separately (≥99.1% nt among them) (Figure 1G,H,L). On the other hand, the NSP3 and NSP4 gene segments exhibited a distinct genetic landscape, with NSP4 shifting from genotype E6 (2012–2013 strains) to E2 (2014 strains) (Figure 1I–K).

Phylogenetic analysis of the NSP1 gene revealed that Brazilian G2P[6] strains from 2012 to 2013 [placed in Lineage R2-IVa] clustered together and showed high nucleotide identity (99.0–99.9% nt) with traditional G2P[4] strains detected worldwide (2005–2015), G2P[4] strains documented in Brazil in 2008–2010 (99–99.3% nt), and several uncommon genotypes identified across Asia, Africa and the Americas over the decades (1997–2018) (99–99.6%nt). They were also closely related to emergent DS-1-like double-reassortant strains (G1P[8]; equine-like G3P[8]; bovine-like G8P[8]) reported globally between 2012 and 2018 (99–99.3% nt), including Brazilian representatives (RVA/Human-wt/BRA/IAL-R3122/2013/G1P[8]; RVA/Human-wt/BRA/LVCA_30390/2019/G6P[8]; RVA/Human-wt/BRA/IAL-R193/2017/G8P[8]) (99.0–99.2% nt). Conversely, the IAL 2014 G2P[6] strains [classified within Lineage R2-IVa] showed similarity in NSP1 with classical G2P[4] strains from Australia and Belgium, detected in 2006, and non-classical G12P[6]/G3P[4] strains reported in Thailand in 2012, Hungary in 2012 and Brazil in 2019 [LVCA_30413; ON331602] (99–99.3% nt). As expected, these strains were genetically related to G1P[8]/G6P[8]/G8P[8] DS-1-like reassortant strains identified in Vietnam (RVA/Human-wt/VNM/SP026/2012/G1P[8]) and Brazil (RVA/Human-wt/BRA/LVCA_30390/2019/G6P[8]; RVA/Human-wt/BRA/IAL-R193/2017/G8P[8]) (99–99.1% nt). Both clusters shared high nucleotide identity (99–99.9% nt) with a common set of reference strains from diverse genotypes and regions, including G2P[4] (AUS 2006, BEL 2006), G12P[6] (THA 2012, BRA 2019), G1P[8] DS-1-like (VNM 2012), G3P[4] (HUN 2012), G6P[8] and G8P[8] (BRA 2017–2019) (Table 1, Figure 1G). The IAL-R1175/12 strain was excluded from the NSP1 segment analyses due to its very short sequence, which was interfering with the alignments and phylogenetic inferences.

In relation to the NSP2 gene, strains IAL-R1000/2012, IAL-R1007/2012, IAL-R1142/2012, IAL-R1228/2012, IAL-R1175/2012 and IAL-R126/2013 [grouped within Lineage N2-V] showed high nucleotide identity (≥99.2% nt) with traditional G2P[4] strains from India, Malawi and Zambia (2012–2015), and with unusual G2P[8]/G9P[4] strains from Zambia, USA and India (2008–2014) (≥99.3% nt). They also shared similarity (99.6–99.9% nt) with double-reassortant G1P[8] DS-1-like strains from Malawi (2013). Strains IAL-R50/2014 and IAL-R52/2014 [assigned to Lineage N2-V] exhibited 99.0–99.4% nt identity with G2P[4] strains from Italy, Belgium, South Korea and Brazil (2006–2011). These strains also showed genetic relationship (99.0–99.1% nt) to atypical G2P[8] and G3P[6] genotypes from India and the Dominican Republic (2016), and with double-reassortant G1P[8] DS1-like and equine-like G3P[8] DS1-like strains (99.0–99.7% nt) recognized internationally (2012–2015), including Brazilian strains from 2013 (IAL-R3123/G1P[8]) and 2016 (IAL-R645_Yarah/G3P[8]) (99.0–99.4% nt) (Table 1, Figure 1H).

As for the NSP3 gene, phylogenetic analysis indicated that strains IAL from 2012–2013, belonging to Lineage T2-V (≥99% nt among them), formed a well-supported cluster with standard G2P[4] strains circulating between 2005 and 2021 in Africa, Asia, Oceania, Europe and North America continents (≥99.0% nt), and were also homologous to Brazilian G2P[4] strains from 2008–2014 (99.0–99.5% nt). These strains further showed high similarity (99.0–99.7% nt) to atypical genotypes (G2P[8]; G3P[6]; G12P[6]; G9P[4]; G2G12P[4]P[8]) identified in the USA, Ethiopia, Bangladesh, India and Guatemala (2000–2016), aligned with strain RVA/Human-wt/BRA/TO-251/2010/G8P[4] (99.0–99.1% nt), and were related to G1P[8] DS-1-like and equine-like G3P[8] DS-1-like strains from India (2017) and Russia (2021) (99.1–99.5% nt). Brazilian IAL 2014 strains clustered separately from each other (shared 98.3% nt identity). Strain IAL-R52/2014 [Lineage T2-V] grouped with G2P[4] strains from 1999–2011 in Mauritius, India, Bangladesh, Australia, Italy, USA and Brazil (2008–2013) (99.0–99.3% nt), and was also similar (9.2–99.3% nt) to non-classical genotypes (G2P[4]P[8]; G3P[6]; G12P[6]; G1G4P[4]) reported during 1999–2009 in the USA, Ethiopia, Bangladesh, Paraguay and Brazil (RVA/Human-wt/BRA/TO-251/2010/G8P[4]) (99.0% nt). Meanwhile, strain IAL-R50/2014 [Lineage T2-V] clustered exclusively with G2P[4] strains from Asia (2013–2015) (99.0–99.2% nt), and showed 99.1–99.3% nt similarity with non-classical G6P[4]/G3P[4]/G2P[8]/G3P[6] strains circulating in Australia, Hungary, the Dominican Republic and India (2008–2016). It also exhibited high identity (99.0–99.8% nt) with emergent double-reassortant G1P[8]/G3P[8]/G8P[8] strains detected globally (2012–2017), including Brazilian strains IAL-R3122/2013/G1P[8], IAL-R3165/2013/G1P[8] and IAL-R330/2015/G3P[8] (99.2–99.6% nt). In the NSP3 gene, a high nucleotide identity (99–100%) was also observed between the IAL 2012–2013 and 2014 strains, which shared a common set of global reference strains, including G2P[4] strains from Mauritius, India (2005–2007, 2011), the USA (2008), Bangladesh (2005, 2008), Australia (2010), Italy (2008) and Brazil (2008–2013); G3P[6] from Ethiopia (2009); G8P[4] from Brazil (2010); G9P[4] from India (2011); and G12P[6] from Bangladesh (2000) (Table 1, Figure 1I).

The Brazilian strains IAL-R50/2014 and IAL-R52/2014, classified within the NSP4 E2-VI lineage, exhibited elevated nucleotide similarity (99.0–99.9% nt) with a wide range of classical (G2P[4]), atypical (G1P[6]; G3P[6]) and double-reassortant (G1P[8] DS-1-like; equine-like G3P[8] DS-1-like) strains detected across the globe between 2005 and 2021, including previously reported Brazilian strains (RVA/Human-wt/BRA/IAL-R594/2015/G3P[8]; RVA/Human-wt/BRA/IAL-R608/2015/G3P[8]; RVA/Human-wt/BRA/IAL-R330/2015/G3P[8]; RVA/Human-wt/BRA/IAL-R3123/2013/G1P[8]; RVA/Human-wt/BRA/AM-17-338/2017/G3P[6]) (99.1–99.9% nt). These IAL 2014 E2-VI strains exhibited 100% nt identity among themselves and clustered distinctly from the G2P[6] strains linked to the 1997 neonates’ outbreak from Belém [42], which belong to Lineage IV [NB334; NB140] (Table 1, Figure 1J). Strains IAL-R1000/2012, IAL-R1007/2012, IAL-R1142/2012, IAL-R1228/2012, IAL-R1175/2012 and IAL-R126/2013 (≥99.8% nt between them) are members of the rare NSP4 E6 genotype, for which no lineages have been defined so far, and exhibited high nucleotide identity with classical G2P[4]/G2P[x] strains (≥99.7% nt) from India (2011–2012), as well as with uncommon or not-typed G9P[4]/G9P[x]/G[x]P[x]/G12P[4]/G12P[6] strains (≥99.% nt) from India, Ghana, USA, Denmark, Bangladesh, Mozambique, Russia and Benin (2000–2022). Among the strains showing 100% nucleotide identity, the most notable include G9P[4] strains from India (2010–2015), Ghana (2015–2016) and the USA (2010), as well as G2P[x] (2012), G9P[x] (2011), GxP[x] (2012), G12P[4] (2015) and G2P[4] strains (2012), with all of them identified in India (Table 1, Figure 1K).

Lastly, NSP5 gene analysis revealed that strains IAL-R1000/2012, IAL-R1007/2012, IAL-R1142/2012, IAL-R1228/2012, IAL-R1175/2012 and IAL-R126/2013 exhibited high nucleotide identity (99.0–99.8%) with typical G2P[4] strains widespread across Africa, Asia, the Americas and Oceania from 2008 to 2014. They also displayed significant similarity (99.2–99.6% nt) with Brazilian G2P[4] strains (2008–2014) and atypical G9P[4]/G2P[4]P[8]/G3P[6]/G2G12P[4]P[8] genotypes identified in Mozambique, the USA, Ethiopia and Guatemala (2008–2017). Moreover, these strains clustered with emerging double-reassortant DS-1-like G1P[8] and equine-like G3P[8] DS-1-like strains from Malawi and China (2013–2020), with nucleotide identity ranging from 99.5% to 99.8%. Strains IAL-R50/2014 and IAL-R52/2014 showed high nucleotide identity (99.2–99.6%) with classical G2P[4] strains from 2003–2015 across Paraguay, South Africa, Europe, Asia and Australia. They were also closely related (99.0–99.6% nt) to diverse atypical genotypes (G6P[6]; G6P[4]; G1G4P[4]; G2P[8]; G8P[4]; G3P[4]; G29P[6]; G3P[6]; G9P[6]) reported between 1999 and 2016 worldwide and highly similar (99.1–99.8% nt) to unusual Brazilian strains (G8P[4]; G12P[6]; G6P[8]; G3P[6]) from 2010 to 2019. Furthermore, these strains clustered with emerging double-reassortant G1P[8]/G3P[8]/G8P[8] strains circulating globally from 2012 to 2021, including Brazilian isolates (RVA/Human-wt/BRA/IAL-R3123/2013/G1P[8]; RVA/Human-wt/BRA/IAL-R594/2015/G3P[8]; RVA/Human-wt/BRA/IAL-R645_Yarah/2016/G3P[8]; RVA/Human-wt/BRA/IAL-R193/2017/G8P[8]) (99.0–99.8% nt) (Table 1, Figure 1L).

Interestingly, the IAL 2014 NSP5 strains exhibited high nucleotide similarity to G2P[6] strains detected in the USA in 2006 [06-242] (99–99.1% nt), as well as in the African continent in 1999 [GHA/MRC-DPRU1818], 2009 [SEN/MRC-DPRU2128; MOZ/MAN-302535] and 2015 [MOZ/HGQ0561] (99–99.3% nt). They also showed close similarity to the mixed RVA/Human-wt/ZAF/MRC-DPRU228/2009/G1G2P[6] strain (99.0–99.1% nt). Notably, both groups of strains (IAL 2012–2013 and IAL 2014) showed high similarity with the RVA/Human-tc/USA/Ph158/1998/G9P[6] strain, with nucleotide identities of 99.2–99.4% for the 2014 strains and 99.0–99.1% nt for the 2012–2013 strains. A comparable pattern was observed in the VP6, NSP1 and NSP3 genes, reinforcing the genetic consistency across these segments (Figure 1L).

## 4. Discussion

This study provides a comprehensive 11-segments analysis of eight G2P[6] RVA strains identified in Brazil between 2007 and 2014. Given the limited availability of sequencing data for RVA G2P[6] strains [33,42,58,59,60], the findings herein provide valuable insights into the genomic diversity and evolutionary dynamics of this uncommon genotype, particularly regarding strains of American origin.

Phylogenetic reconstructions exposed the existence of two genetically distinct clusters: strains from 2012–2013 displaying I2-R2-C2-M2-A2-N2-T2-E6-H2 constellation, and strains from 2014 exhibiting a pure DS-1-like genomic backbone (I2-R2-C2-M2-A2-N2-T2-E2-H2). The vast majority of G2P[6] strains deposited in GenBank are associated with the conserved DS-1-like constellation (I2-R2-C2-M2-A2-N2-T2-E2-H2), predominantly of African origin [58,59,60], with only a single representative strain from the Americas (RVA/Human-wt/USA/06-242/2006/G2P[6]) having all 11 genomic segments sequenced [33]. This same genetic backbone was also observed in the Brazilian 2014 strains characterized in this study (IAL-R50 and IAL-R52), further supporting this common association.

On the other hand, the Brazilian 2012–2013 strains exhibited the rare E6 genotype (I2-R2-C2-M2-A2-N2-T2-E6-H2), which, to date, has not been reported in association with the G2P[6] genotype. The uncommon E6 genotype was first identified in a human G8P[6] strain in India in 2000 and was subsequently detected in G12P[6] strains in Bangladesh (2000–2001) and in a G12P[9] strain in Italy (2012) [61,62,63]. Studies suggest a possible animal origin for the E6 genotype [62,64]. The I2-R2-C2-M2-A2-N2-T2-E6-H2 genomic constellation appears to be more frequently linked to the G9P[4] genotype, as reported in several regions, including Latin America [35,65,66,67]. It has been proposed that this constellation emerged through multiple reassortment events involving DS-1-like strains and other strains donating the NSP4 segment (E6) [66]. Similar reassortment events may have occurred between the Brazilian G2P[6] strains identified in 2012–2013 and other co-circulating strains. However, the phylogenetic analyses conducted in this study were unable to confirm such events, likely due to the limited availability of full-genome sequences from Brazilian strains, particularly atypical G9P[4] genotypes. These findings underscore the need to expand the full-genome sequencing of RVA strains in Brazil, especially for rare or unusual genotypes [68,69,70]. Interestingly, G9P[4]-E6 strains, which were frequently detected between 2009 and 2016, declined after 2017, and the E6 genotype has not been reported since [64]. Recent data from Iran (2021–2022) and the Czech Republic (2018) indicate the replacement of E6 by E2 in G9P[4] strains [71,72]. Extrapolating this trend to our genetic findings, it is plausible that a similar replacement occurred in the Brazilian IAL G2P[6] strains detected in 2014, which also presented the NSP4-E2 genotype instead of E6.

The G2 VP7 gene evolved through successive lineage replacements, reflecting genetic changes and possible reassortment events over time, with its evolutionary history indicating diversification into five major lineages (I–V) [57,73,74]. G2 strains were initially dominated by Lineages I–III and gradually replaced by Lineage IVnon-a between 1985 and 1996. From 1999 onward, a clear shift toward Lineage IVa occurred, which has remained globally dominant since the early 2000s [57,73,74]. Although G2-IVnon-a variants are less common, both sub-lineages have been reported to co-circulate, suggesting concurrent transmission in some regions [21,75,76]. Lineage V emerged around 2005–2006 and now predominates among modern G2 strains [77]. Interestingly, its antigenic profile is similar to G2-IVnon-a strains, suggesting that its emergence was likely due to intragenotypic reassortment rather than antigenic escape [57]. A phylogenetic analysis of eight Brazilian IAL G2P[6] strains showed that six (IAL-R1000/2012, IAL-R1007/2012, IAL-R1142/2012, IAL-R1228/2012, IAL-R1175/2012 and IAL-R126/2013) clustered within Lineage IVa, while strains IAL-R50/2014 and IAL-R52/2014 belonged to VP7 G2 Lineage V. These findings align with the global evolutionary pattern of the G2 VP7 gene, indicating coexistence and possible transition between these lineages in the Brazilian context as well.

Phylogenetic analysis of the VP4 gene in Brazilian G2P[6] strains revealed their affiliation with the P[6]-Ia lineage, alongside globally circulating human genotypes such as G12P[6], G1P[6], G9P[6], G8P[6], G4P[6], G3P[6] and G6P[6] [70,78,79,80,81]. The P[6]-Ia lineage also includes porcine and porcine-like human strains reported worldwide [39,82,83]; nevertheless, the IAL strains examined in this study showed no evidence of zoonotic origin. Additionally, the Brazilian IAL P[6] strains segregated into two clusters: those from 2012–2013 grouped with African and Asian strains, while the 2014 strains clustered exclusively with Asian lineages. This phylogenetic pattern reflects the complex geographic spread and epidemiological dynamics of the P[6]-Ia lineage. Compared to earlier Brazilian P[6] RVA strains, the sequences analyzed here exhibited greater genetic divergence [42,70,84,85], indicating likely independent introductions into Brazil, possibly from Africa and Asia. This also suggests gaps in local surveillance, which may have impaired the detection of previously circulating Brazilian strains.

Considering VP7 and VP4 together, further functional studies focusing on amino acid variations involved in antigenicity and host interactions, given their demonstrated roles in modulating viral fitness, immune evasion, and transmissibility through changes in antigenic epitopes and host cell binding, could provide additional insights into potential differences in these viral properties [36,69,70,73].

Phylogenetic analyses of the 11 gene segments of Brazilian G2P[6] strains revealed the presence of two temporally and genetically distinct groups. Group 1, comprising strains detected between 2012 and 2013 (IAL-R1000/2012, R1007/2012, R1142/2012, R1228/2012, R1175/2012, and R126/2013), showed high internal similarity and was clearly divergent from Group 2, represented by the 2014 strains (IAL-R50/2014 and IAL-R52/2014), which also clustered separately, with high nucleotide identity between them.

Group 1 (strains 2012–2013) clustered with globally distributed classical G2P[4] strains circulating between 2005 and 2021 [21,36,86,87]. The IAL G2P[6] 2012–2013 strains were strongly related to earlier Brazilian G2P[4] strains (2008–2014) [88,89], as well as to atypical genotypes such as G9P[4], G3P[4] and G12P[6] identified across multiple continents [65,66,84,90]. They also showed similarities to DS-1-like double-reassortant strains, including equine-like G3P[8], G1P[8] and G8P[8] strains circulating in Africa, Asia, Europa, North America and Latin America, including Brazil, between 2012 and 2021 [59,69,91,92]. Notably, they harbored the rare NSP4 E6 genotype, which has been associated with emergent strains in distinct geographic contexts and showed high nucleotide identity with strains from Asia and Africa [64,93]. The genetic similarities with strains from diverse continents highlight the existence of a highly dynamic and interconnected global viral pool, marked by the circulation of both human and potentially zoonotic lineages, positioning Brazil as a potential hotspot for genetic reassortment and recombination.

Group 2 (strains 2014) appeared to be associated with emergent DS-1-like genetic constellations. The presence of the NSP4 E2-IV lineage, frequently found in recently emerging DS-1-like G1P[8] and equine-like G3P[8] strains, supports this hypothesis [94,95,96]. The IAL G2P[6] 2014 strains were clearly genetically related to emergent DS-1-like double-reassortant strains, predominantly detected in Asia, as well as to previously reported Brazilian strains [96,97,98,99]. In addition, although to a lesser extent than their similarity with emergent DS-1-like strains, they also exhibited relatedness to classical G2P[4] strains and atypical genotypes such as G9P[4] and G3P[6] [58,85,86,90,97]. Their robust genetic similarity to Brazilian DS-1-like double-reassortant strains identified in 2013 and 2016 [96,99] suggests that the IAL G2P[6] 2014 strains were rapidly integrated into the local viral landscape. This finding highlights the adaptability of emergent DS-1-like strains and their ability to establish themselves in new epidemiological settings through reassortment with locally circulating strains, underscoring 2014 as a pivotal year in the local diversification of G2P[6] variants.

Furthermore, the lack of phylogenetic continuity between the studied Brazilian G2P[6] strains and previously reported strains from Brazil, North America or Africa [33,42,59,60] reinforces the hypothesis of multiple independent introduction events into Brazil, likely through distinct routes involving Africa, Asia and Latin America. The consistent binary clustering pattern observed across multiple structural and non-structural gene segments indicates that the observed diversity results from both viral migration and local reassortment events involving international and regional lineages. This highlights the dynamic interplay between viral dispersal and genome plasticity in shaping the local viral population.

A noteworthy point is that the DS-1-like constellation identified in the IAL G2P[6] strains shows a strong genetic connection with emerging intergenogroup DS-1-like human strains, such as G1/G3/G8P[8], reported globally, including in Brazil [59,69,91,92,96,97,98,99]. In contrast, the rare G12P[6] DS-1-like strains detected during 2011–2020 and recently analyzed at the full-genome level in Brazil [70], show no genetic evidence of relatedness to these emergent intergenogroup DS-1-like strains. The distinct evolutionary trajectories observed for the two P[6]-bearing strains, G2P[6] and G12P[6], despite their sharing nearly identical DS-1-like genomic backbones, underscore the complex interplay of selective pressures shaping RVA evolution. Although both strains circulate under similar ecological conditions in Brazil and contribute minimally to the RVA-associated diarrheal disease burden, with limited detection rates, factors beyond genomic constellation, such as host immunity, viral fitness and ecological competition, play a critical role in determining their persistence and dissemination.

## 5. Conclusions

In conclusion, the Brazilian G2P[6] strains analyzed in this study reflect a complex evolutionary and epidemiological dynamic, originating from multiple genetic sources and showing links to both classical and emerging strains. These findings reveal a scenario characterized by multiple reassortment events and extensive international circulation, underscoring the highly dynamic nature of the G2P[6] genotype and its strong potential for evolution. The genetic relatedness to strains from diverse geographic regions suggests that factors such as population mobility, international trade, and migration flows have influenced the introduction and spread of these strains in Brazil. No clear genomic or phylogenetic differences explaining why G2P[4] holds greater epidemiological importance compared to G2P[6] could be identified. Both genotypes share DS-1-like constellations, exhibit multiple introductions of distinct RVA strains into the human population, and display genetic diversification. It was not possible to pinpoint a specific genetic or evolutionary factor underlying the higher epidemiological relevance of G2P[4] in diarrheal disease. Collectively, these data emphasize the importance of ongoing molecular surveillance of RVA diversity, particularly focusing on variants with potential epidemiological impact.

## Figures and Tables

**Table 1:** Demographic and spatial data, migration profile of dsRNA segments, genotype constellation and lineages of RVA G2P[6] strains, Brazil, 2007–2014. The rare genotype E6-NSP4 and common genotype E2-NSP4 are highlighted in lilac.

Strain	Age	Gender	City	State	Profile	VP7	VP4	VP6	VP1	VP2	VP3	NSP1	NSP2	NSP3	NSP4	NSP5
						G2	P[6]	I2	R2	C2	M2	A2	N2	T2	E6	H2
RVA/Human-wt/BRA/IAL-R1000/2012/G2P[6]	2 months	F	São Paulo	SP	Short	IVa	I-a	V	V	IVa	V	IVa	V	V	-	IVa
RVA/Human-wt/BRA/IAL-R1007/2012/G2P[6]	4 months	F	São Paulo	SP	Short	IVa	I-a	V	V	IVa	V	IVa	V	V	-	IVa
RVA/Human-wt/BRA/IAL-R1142/2012/G2P[6]	2 months	M	São Paulo	SP	Short	IVa	I-a	V	V	IVa	V	IVa	V	V	-	IVa
RVA/Human-wt/BRA/IAL-R1175/2012/G2P[6]	1 year	F	São Paulo	SP	Short	IVa	I-a	V	V	IVa	V	-	V	V	-	IVa
RVA/Human-wt/BRA/IAL-R1228/2012/G2P[6]	10 months	M	Nova Laranjeiras	PR	Short	IVa	I-a	V	V	IVa	V	IVa	V	V	-	IVa
RVA/Human-wt/BRA/IAL-R126/2013/G2P[6]	4 years	F	São Paulo	SP	Short	IVa	I-a	V	V	IVa	V	IVa	V	V	-	IVa
**Strain**	**Age**	**Gender**	**City**	**State**	**Profile**	**VP7**	**VP4**	**VP6**	**VP1**	**VP2**	**VP3**	**NSP1**	**NSP2**	**NSP3**	**NSP4**	**NSP5**
						**G2**	**P[6]**	**I2**	**R2**	**C2**	**M2**	**A2**	**N2**	**T2**	**E2**	**H2**
RVA/Human-wt/BRA/IAL-R50/2014/G2P[6]	26 days	U	São Paulo	SP	Short	V	I-a	V	V	IVa	V	IVa	V	V	VI	IVa
RVA/Human-wt/BRA/IAL-R52/2014/G2P[6]	22 days	M	São Paulo	SP	Short	V	I-a	V	V	IVa	V	IVa	V	V	VI	IVa

SP: São Paulo state; PR: Paraná state; M: male; F: female; U: unidentified.

## Data Availability

Nucleotide sequences from this study were submitted to GenBank with accession numbers: NSP1: PV126468–PV126475; NSP2: PV126476–PV126483; NSP3: PV126484–PV126491; NSP4: PV299264–PV299271; NSP5: PV335520–PV335527; VP1: PV299272–PV299279; VP2: PV335407–PV335414; VP3: PV335415–PV335422; VP4: PV335423–PV335430; VP6: PV335644–PV335651; and VP7: PV335652–PV335659.

## Supplementary Materials

The following supporting information can be downloaded at: https://www.mdpi.com/article/10.3390/v17081103/s1. Table S1: Length and nucleotide position of each gene segment of the Brazilian RVA G2P[6] strains detected in Brazil, 2007–2014.
