# Supplementary material for: Unveiling the Genomic Landscape of G2P[6] Rotavirus a Strains in Brazil: Evolutionary and Epidemiological Perspectives
Source: Viruses. 2025 Aug 11;17(8):1103. doi: 10.3390/v17081103 (PMC12390652; doi:10.3390/v17081103)
Supplement: Supplementary file 1 [file viruses-17-01103-s001.zip › viruses-3783047-supplementary.pdf]

Supplement 1. Length and nucleotide position of each gene segment of the Brazilian RVA G2P[6] strains detected in Brazil, 2007-2014.

				**IAL-R1000/2012**		**IAL-R1007/2012**		**IAL-R1142/2012**		**IAL-R1175/2012**		**IAL-R1228/2012**		**IAL-R126/2013**		**IAL-R50/2014**		**IAL-R52/2014**	
**Gene segment**	**Size (nt)**	**Encoded protein**	**ORF position (nt)**	**Size (nt)**	**Position (nt)**	**Size (nt)**	**Position (nt)**	**Size (nt)**	**Position (nt)**	**Size (nt)**	**Position (nt)**	**Size (nt)**	**Position (nt)**	**Size (nt)**	**Position (nt)**	**Size (nt)**	**Position (nt)**	**Size (nt)**	**Position (nt)**
1	3,302	VP1	19-3,285	517	73-589	601	73-673	627	10-636	609	68-676	665	11-675	643	32-674	607	71-677	664	11-671
2	2,690	VP2	17-2,656	520	84-603	519	101-619	520	100-619	554	54-607	572	40-611	564	44-607	553	56-608	557	54-610
3	2,591	VP3	50-2,557	572	45-616	469	130-598	496	124-619	407	112-518	612	10-621	565	10-574	636	10-645	624	12-635
4	2,362	VP4	10-2,337	743	94-836	751	99-849	742	106-847	630	134-763	739	99-837	726	110-835	695	53-793	793	104-798
5	1,611	NSP1	11-1,492	1490	3-1492	1486	3-1488	1392	97-1488	398	58-979	1484	3-1486	1270	3-1272	1434	3-1436	1494	3-1496
6	1,356	VP6	24-1,217	1281	12-1292	1319	12-1328	1320	12-1328	1276	12-1287	1319	12-1328	1206	37-1242	1225	60-1284	1277	9-1318
7	1,104	NSP3	16-948	945	61-1005	942	5-946	1005	2-1006	776	16-791	983	16-998	912	94-1005	931	969-1026	968	16-983
8	1,059	NSP2	30-983	973	25-997	955	29-983	955	29-983	862	1-844	950	29-978	950	29-978	949	29-977	862	29-970
9	1,062	VP7	49-1,029	883	62-944	920	31-950	873	60-932	955	1-951	901	46-946	954	17-970	969	1-967	914	26-939
10	751	NSP4	42-569	590	53-642	531	95-625	655	1-649	586	27-612	529	97-625	450	173-622	690	1-677	714	1-707
11	816	NSP5/6	5-598	767	12-778	767	12-778	706	69-774	659	70-728	767	12-778	746	12-757	685	70-754	767	12-778
**Total genome**	**16,283**	-	-	**9,281**	-	**9,260**	-	**9,291**	-	**7,712**	-	**9,521**	-	**8,986**	-	**9,374**	-	**9,634**	-
